# Micro-nutrient sufficiency in mothers and babies: management of deficiencies while avoiding overload during pregnancy

**DOI:** 10.3389/fnut.2025.1476672

**Published:** 2025-04-01

**Authors:** Noor Fatima, Sanabil Yaqoob, Laraib Rana, Aysha Imtiaz, Muhammad Jawad Iqbal, Zahid Bashir, Amal Shaukat, Hiba Naveed, Waleed Sultan, Muneeba Afzal, Zara Kashif, Fahad Al-Asmari, Qing Shen, Yongkun Ma

**Affiliations:** ^1^NIFSAT - National Institute of Food Science and Technology, University of Agriculture, Faisalabad, Pakistan; ^2^Laboratory of Food Nutrition and Clinical Research, Institute of Seafood, Zhejiang Gongshang University, Hangzhou, China; ^3^Department of Food Science and Technology, Faculty of Science and Technology, University of Central Punjab, Lahore, Pakistan; ^4^School of Food and Biological Engineering, Jiangsu University, Zhenjiang, China; ^5^School of Food Science and Engineering, Yangzhou University, Yangzhou, China; ^6^School of Food Science and Technology, Minhaj University, Lahore, Pakistan; ^7^Department of Food and Nutrition Sciences, College of Agriculture and Food Sciences, King Faisal University, Hofuf, Saudi Arabia

**Keywords:** pregnancy, micronutrients, supplementation, fetal growth, Iron, folic acid, calcium, iodine

## Abstract

Pregnancy is a period characterized by extensive physiological changes in both the mother and fetus. During this period, the nutritional status of the mother has a profound and irreversible impact on her health and the growth and development of the fetus. The fetus depends exclusively on the mother and drives nutrients through the placenta. Therefore, mothers must be provided with a well-balanced diet that is adequate in both macro- and micronutrients. Most pregnant women generally manage to get adequate macronutrients; however, many women fail to get micronutrients up to the recommended dietary allowance. Micronutrients such as vitamins and minerals are necessary for preventing congenital abnormalities and the optimal development of the brain and body of the fetus. Their inadequacy can lead to complications like anemia, hypertension, pre-eclampsia, maternal and fetal hypothyroidism, premature infants, intrauterine growth restriction, stillbirth, and other negative pregnancy outcomes. New studies recommend the use of prenatal micronutrient supplements to prevent birth defects and health issues caused by deficiencies in folic acid, iron, iodine, and calcium during pregnancy. This is especially important in developing nations where deficiencies are prevalent. Also while using these supplements, their upper limits (UL) must be considered to avoid overload. In this review, we provide an overview of the four most critical micronutrients during pregnancy: iron, folic acid, iodine, and calcium. We provide insight into their sources, RDAs, deficiency consequences, and the need for supplementation while considering the risk of micronutrient overload. To maximize the potential benefits while minimizing the risk of nutrient overload, although knowledge gaps remain.

## Graphical abstract

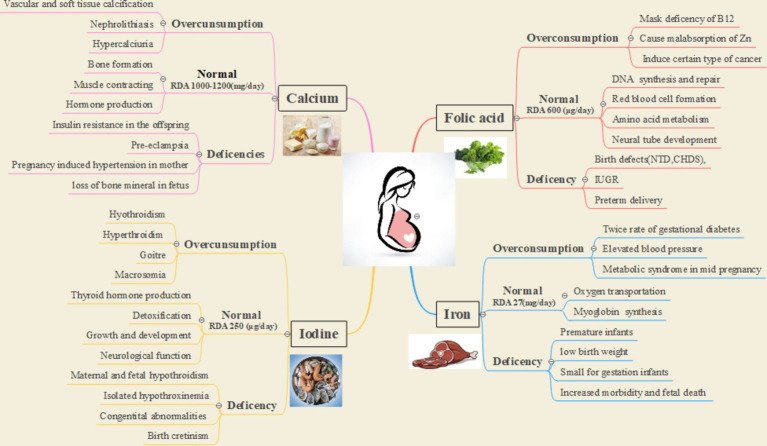

## 1 Introduction

Pregnancy is a period of rapid and extensive physiological changes that increase maternal nutritional requirements (1, 2). The first 1,000 days of a newborn’s life (from gestation to second birthday) are considered the “golden opportunity” for determining their health in life (3). Therefore, the nutritional status of the mother before and during pregnancy has a significant and irreversible impact on the growth of infants including birth weight, as well as the mother’s health (4). Adequate macronutrients and micronutrients are mandatory to promote healthy gestation. Their deficiency can trigger a state of biological competition between the mother and infant (5), leading to complications such as anemia and hypertension, as well as impaired function, growth, and development in the infant. In addition to negative pregnancy outcomes, micronutrient deficiencies can also affect adulthood, intergenerational health, morbidity, and infant mortality (6, 7).

Every 2 minutes, a woman dies during pregnancy or childbirth, revealing alarming setbacks for women’s health in recent years. Among the most essential micronutrients, the four most vital ones are (1) iron, (2) folic acid/B9, (3) calcium, and (4) iodine (see graphical abstract). Iron is the most critical nutrient in the body because it helps in the production of hemoglobin and myoglobin and the metabolism of proteins (8–10). Insufficient iron intake can cause anemia and iron deficiency anemia. Globally, iron deficiency is affecting about 45 million pregnant women (11–13). That is why the idea that iron supplementation should be universal during pregnancy is under discussion (14–16). However, iron supplementation can also cause some gastrointestinal problems, like nausea (17). Folic acid is involved in the development of neural tube and protein metabolism and prevents the development of birth defects in the fetus, pre-eclampsia, spontaneous miscarriage, and placental anomalies in the mother (18–21). Despite the benefits of folic acid supplementation, its overconsumption can cause some adverse effects like increased insulin resistance and low levels of adiponectin in children, leading to childhood obesity (22). Adequate calcium intake is crucial for fetal bone development and reducing risks of preeclampsia and preterm delivery, which is why its intestinal absorption doubles in pregnancy (23–25). Hypocalcemia is not very common during pregnancy but occurs due to hypoparathyroidism and extremely low dietary calcium intake (26, 27). On the other hand, excessive calcium supplementation can lead to hypercalcemia and kidney stone risk (28, 29). Iodine is essential for thyroid hormone production, particularly during pregnancy due to increased maternal thyroid demand, a higher glomerular filtration rate, and fetal needs (30–35). According to the International Council for Control of Iodine Deficiency Disorders (ICCIDD), and the World Health Organization (WHO), the iodine requirement for pregnant and lactating women is 250 μg/day. However, excessive iodine intake can lead to thyroid disorders in both the mother and fetus, posing risks such as hypothyroidism, goiter, and negative pregnancy outcomes, including macrosomia (36–38). Therefore, women should consume micronutrient dosages according to their own country’s recommendations throughout gestation to support a healthy pregnancy and fetal growth (Table 1).

**Table 1:** Showing recommended nutrient intake of different countries during pregnancy.

Countries	Iron	Folic acid	Calcium		Iodine	Following
America	27 mg/day (233)	600 mcg /day of dietary folate equivalents (DFEs) (234)	≤ 18 years 19–50 years	1,300 mg/day 1,000 mg/day (235)	220 mcg (236)	National Institute of Health (NIH)
Canada	27 mg/day (237)	600 μg /day of dietary folate equivalents (DFEs) (237)	≤ 18 years 19–50 years	1,300 mg/day 1,000 mg/day (237)	220 μg/day (237)	Health Canada
Australia	27 mg/day (238)	600 μg/day (239)	14–18 years 19–50 years	1,300 mg/day 1,000 mg/day (240)	220 μg/day (241)	National Health and Medical Research Council
New Zealand	27 mg/day (238)	600 μg/day (239)	14–18 years 19–50 years	1,300 mg/day 1,000 mg/day (240)	220 μg/day (241)	National Health and Medical Research Council
Germany	30 mg/day (242)	600 μg/day (242)	<19 years ≥19 years	1,200 mg/day 1,000 mg/day (243)	230 μg/day (244)	German Nutrition Society (DGE) European guidelines
Denmark	26 mg /day (245)	600 μg/day (245)	950 mg /day (245)		200 μg/day (245)	Nordic Nutrition Recommendations
India	27 mg/day (246)	570 μg/day (246)	1,000 mg/day (246)		220 μg/day (246)	Indian Council of Medical Research (ICMR)
France	30 mg/d (247)	_	<19 years ≥19 years	1,200 mg/day 1,000 mg/day (248)	200 μg/day (249)	European Food Safety Authority (EFSA)

Keeping in view the above-given importance of these micronutrients, it is critical to have a deeper understanding of pregnancy nutritional requirements. Therefore, this review provides insights into sources, bioavailability, Recommended Dietary Allowance (RDA), causes and consequences of deficiency, and the need for supplementation while considering the danger of micronutrient overload of the four most vital nutrients during pregnancy: iron, folic acid, iodine, and calcium to assess and improve pregnant women’s health status.

## 2 Iron

In adult individuals, the amount of iron is about 35 to 45 mg per kg of body weight (39–41).

Iron plays a crucial role in the synthesis of myoglobin (in muscle tissue) and hemoglobin. Hemoglobin is used for the transportation of oxygen from the lungs to the tissues through red blood cells (8–10, 39). During pregnancy, iron requirements increase more than twice, particularly in the 2nd and 3rd trimesters. Consequently, the iron RDA for pregnant women is up to 27 mg per day (42–46). Mothers require an increased intake of iron during pregnancy due to three primary factors. Firstly, an increase in the amount of maternal blood and plasma; secondly, a baby demands iron for its current metabolic activities and stores iron reserves for the next 6 months after birth as breastmilk is low in iron; and lastly, the placenta needs a significant amount of iron because of its accelerated metabolic functioning (9, 41, 47–49). Moreover, the daily use of iron during pregnancy is associated with a reduced risk of low birth weight (50). The total iron requirement during pregnancy is expected to be 1,040 mg, with 300 mg for the fetus, 50 mg for the placenta, 450 mg for the increase of maternal red cell mass, and 240 mg for basal iron loss (51).

### 2.1 Sources

There are two different sources of dietary iron heme and no heme. Heme represents 40% of total iron in animal foods and exhibits a bioavailability range of 15 to 40% in the human body, indicating their greater ability to be absorbed and utilized (52). Moreover, non-heme iron is derived from plant-derived sources. Its bioavailability ranges from 1 to 15% and is more susceptible to alteration by various constituents present in food (53). Overall, the most well-known animal source of iron is the liver of the Bovidae family, which includes calf liver as well as liver from pigs, sheep, horses, and ducks. Other animal sources with great iron amounts are the kidney, the brewer’s yeast, and meats (54, 55).

### 2.2 Absorption and factors affecting absorption

**Figure 1:**
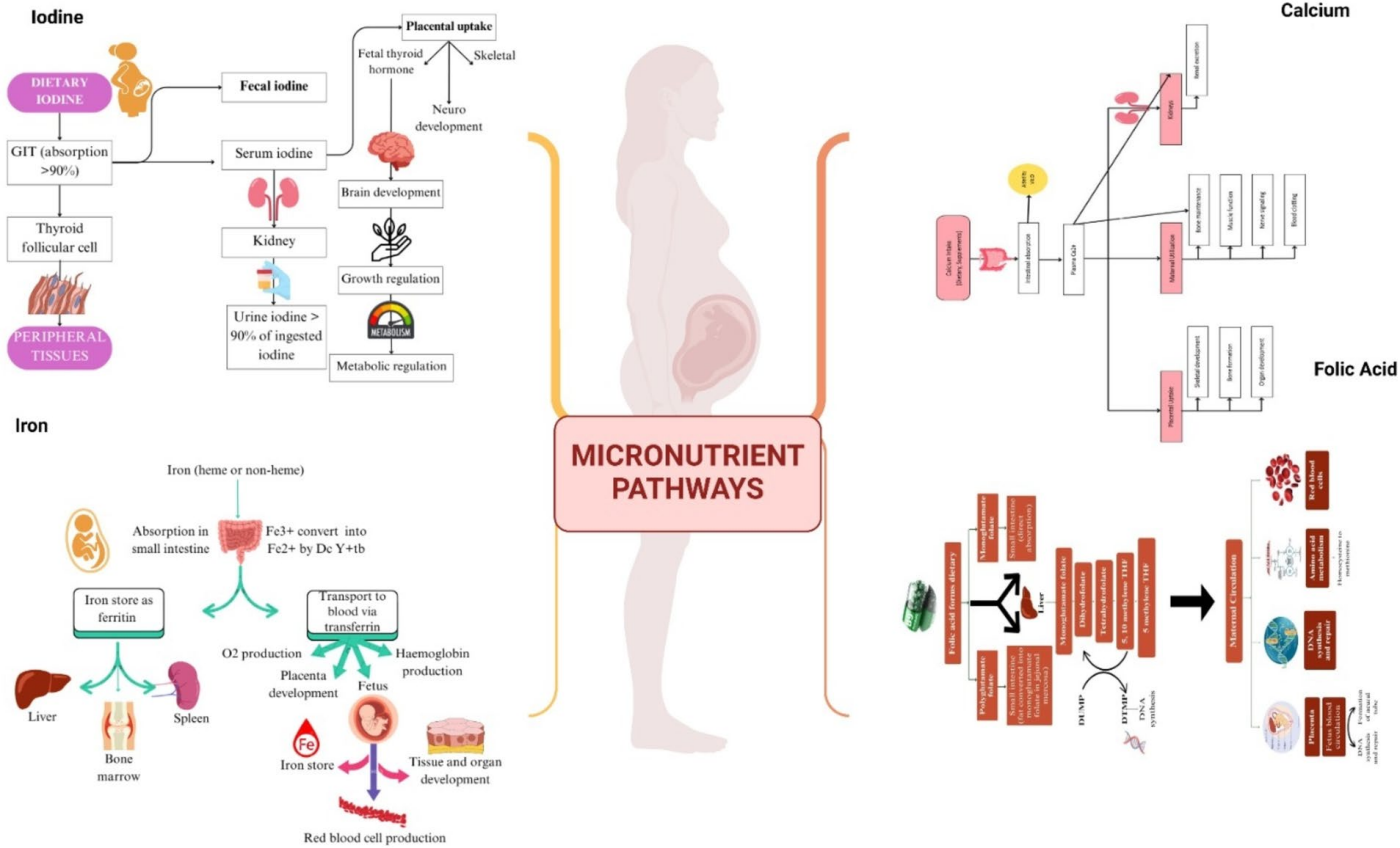
Pathway of absorption of different nutrients during pregnancy.

**Figure 2:**
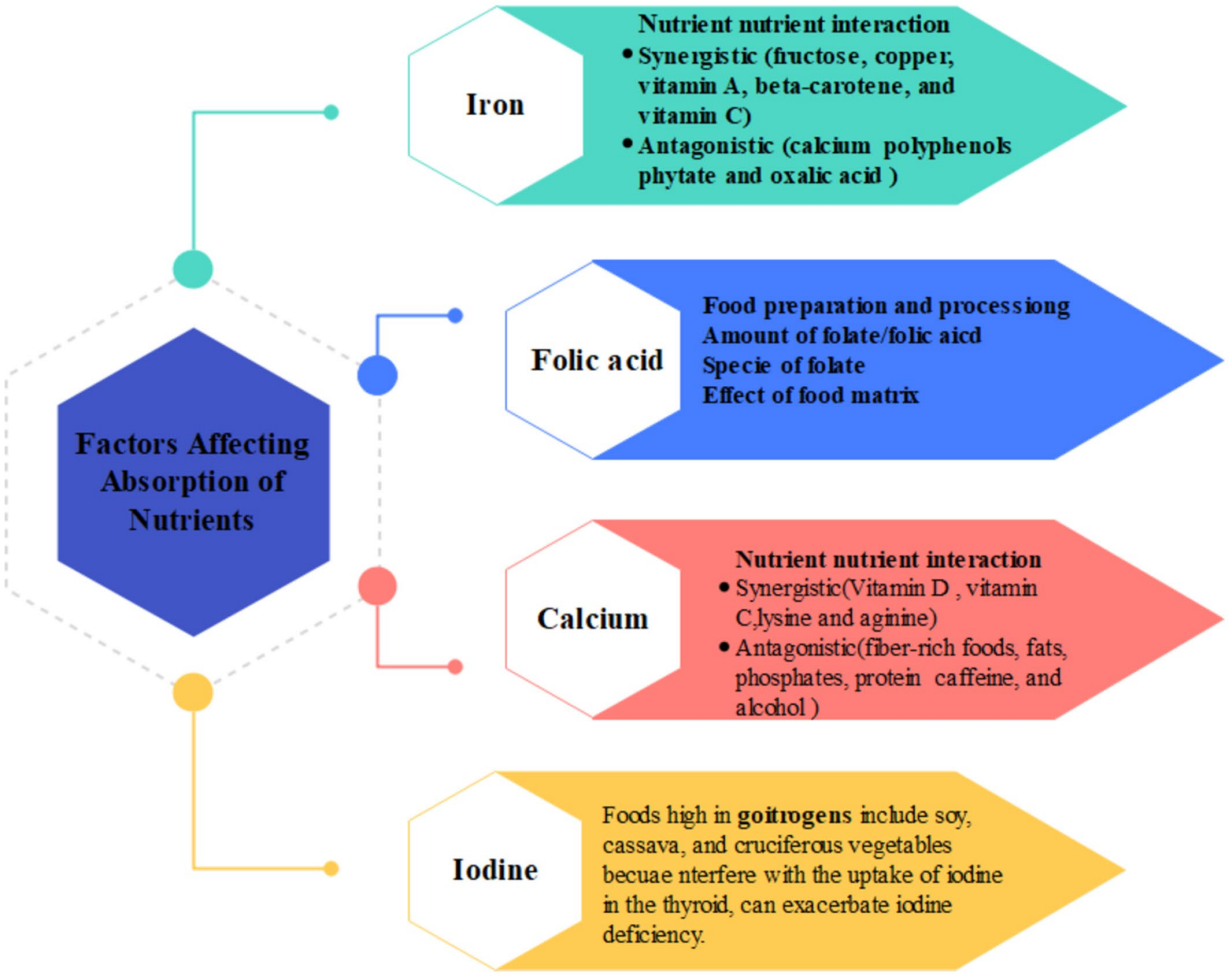
Factors affecting the absorption of different nutrients.

Iron can only be absorbed when it is ferrous (Fe2+) or bound to a protein like heme. In the proximal duodenum, the low pH of gastric acid allows a ferric reductase enzyme, duodenal cytochrome B (Dcytb), to convert insoluble ferric (Fe3+) into absorbable ferrous ions (Fe2+). In enterocytes, iron can be stored as ferritin or transported through the basolateral membrane and into circulation as ferroportin (56) as shown in Figure 1. The bioavailability of dietary iron is not optimal due to potential interactions with other nutrients in food, such as calcium (present in milk-based products like cheese), polyphenols (in caffeine-containing foods like tea and coffee), phytate (in whole-grain-containing products, e.g., bread), and oxalic acid (in beetroot), which decreases iron absorption (57, 58). Conversely, several nutrients, such as fructose, copper, vitamin A, beta-carotene, and vitamin C, have been found to boost the absorption of iron (59). Among these options, vitamin C is the most promising for enhancing the absorption of iron (60). The effect of vitamin C has been proven to be amount-dependent (61, 62) and can increase the absorption of iron only when both nutrients are consumed together (63). Moreover, it has been reported taking 500 mg of ascorbic acid with food increases the absorption of iron six times, but ascorbic acid taken 4–8 h before is less effective (64). Despite stability issues of vitamin C during food preparation, adding vitamin C to the diet appears to be a good way to increase iron consumption (65). However, some studies indicate that the addition of ascorbic acid to the whole diet has no significant impact on increasing iron absorption because of meal composition and the whole food matrix in the diet as shown in Figure 2 (66–69). Furthermore, in order to maximize absorption of oral iron supplements, women should take it on an empty stomach, 1 h before meals, with a source of Vitamin C (ascorbic acid) such as orange. Mothers should also avoid taking other medications such as antacids at the same time because they may impair absorption (70).

### 2.3 Iron deficiency

Iron insufficiency is a prevalent global issue, impacting over 2 billion individuals. This includes approximately 30 % of pregnant women in developed nations, while this prevalence is even higher in underdeveloped nations (8). Women of reproductive age, especially vegans or vegetarians, obese women, those suffering from inflammatory bowel disease (IBD), and endurance athletes are at a higher risk of iron deficiency. Iron insufficiency is the predominant cause of anemia during pregnancy, which affects around 45 million pregnant women globally. Iron deficiency anemia (IDA) is a condition characterized by low levels of hemoglobin. As defined by the WHO, hemoglobin concentrations below 110 g per liter during the 1st and 3rd trimesters or below 105 g per liter during the 2nd trimester are indicative of IDA (11–13, 71–76).

During pregnancy, iron deficiency anemia has adverse effects on both the mother’s and fetus’ well-being. In the fetus, ID and/or IDA have been associated with the risk of premature infants, low birth weight (LBW), or small for gestational age (SGA) infants, and even increased morbidity and fetal death (9, 15, 77). Mothers with iron deficiency might experience difficulties with breathing, fainting, fatigue, palpitations, and sleep disturbances (47–49).

In 2012, the Global Nutrition Target 2025 aimed for a 50% reduction in the prevalence of anemia among women of the reproductive age group (78) Unfortunately, according to the 2021 Global Nutrition Report, there has been limited global progress toward meeting the target. On the contrary, a projected estimate based on the current trends details an expected prevalence in 2025 of more than double the set target level (31.2% rather than 14.3%) (79).

### 2.4 Biomarker

There are several biomarkers available by which we can detect iron deficiency. Hemoglobin levels have been utilized as a means of assessing iron status for centuries, particularly in low-resource nations, due to its simplicity and affordability. Nevertheless, it is deficient in detecting the initial stage of iron insufficiency (80). There are several other iron-specific biomarkers, including serum ferritin, total iron binding capacity saturation (%TSAT), and hepcidin, which can specifically differentiate iron deficiency anemia (IDA) from other causes of anemia (81). All these biomarkers have their pros and cons. as you can see in Table 2.

**Table 2:** Advantages and disadvantages of iron-specific biomarkers.

Biomarkers	Advantage	Disadvantage
Hemoglobin concentration	Its ranges are well-defined and accessible for diagnosis. It is affordability and widespread use make it more useful, particularly in the resource area (80).	It is unable to identify iron deficiency in its early phases even when physiological effects start appearing at the tissue level (80).
Serum ferritin	It helps in the diagnosis of iron deficiency and provides information about the body’s iron reserves (250).	Serum ferritin concentration rises as a result of Infections, inflammation, cardiac failure, renal failure, malignancies, and other conditions that can elevate serum ferritin (251). It does not provide information about how much iron is available for red blood cell production (252).
% Total iron binding capacity saturation (%TSAT)	It helps in the diagnosis of pre-anemic women with iron deficiency (80).	Ferritin is an acute-phase protein, inflammation might increase its levels, while TSAT may be less impacted by inflammation (253).
Serum hepcidin	Depending on inflammation and iron status in the body, it provides insights about who should receive iron supplements (254). It outperforms hemoglobin, serum iron, serum ferritin, TS, and TIBC in the detection of IDA in pregnancy (255).	It is unable to differentiate iron deficiency anemia (IDA) and anemia of chronic disease (ACD) (256).

### 2.5 Supplementation and dosage

Nutritional surveys in the United Kingdom and Norway showed that women’s dietary patterns change little with pregnancy (82, 83). Moreover, only 30 percent of dietary iron can be effectively absorbed even in ideal circumstances, relying solely on dietary iron is not practical for meeting the increased iron requirements during pregnancy. WHO recommends pregnant women to take 30 mg to 60 mg of elemental iron and 400 μg (0.4 mg) of folic acid supplement to prevent maternal anemia, puerperal sepsis, low birth weight, and preterm birth. The equivalent of 60 mg of elemental iron is 300 mg ferrous sulfate heptahydrate, 180 mg ferrous fumarate or 500 mg of ferrous gluconate (84). Preventative iron supplementation during pregnancy leads to a significant reduction of 70% in maternal anemia. Hence, pregnant women should consider the utilization of iron supplements, the appropriate dosage of which should be determined based on the prevalence of maternal anemia within their specific geographic area (14–16). When iron insufficiency is more than 40% of the population WHO recommends 60 mg of daily oral elemental iron and when it is less than 40% WHO recommends a lesser dose of 30 mg daily (85). There are 17 trials (*N* = 24,023) on maternal iron supplementation conducted by the US Preventive Services Task Force. Iron supplementation reduced the risk of maternal iron deficiency anemia at term (4 trials, *n* = 2,230, 8.6% vs. 19.8%) and maternal iron deficiency at term (6 trials, *n* = 2,361, 46% vs. 70%) as compared to placebo or no iron supplement. But were no statistically significant differences in maternal quality of life and maternal health compared with placebo or no supplementation (86). Furthermore, one meta-analysis indicates that intermittent oral iron supplementation with a dose of 120 mg/day is more effective than daily oral iron supplementation with a dose of 60 mg/day in increasing hemoglobin levels among pregnant women, with lesser side effects. Crucially, this approach is linked with a significant reduction in adverse effects related to iron supplementation. That is why intermittent oral iron supplementation is recommended for those individuals who are not able to adhere to the daily regime due to adverse events (87).

However, women with severe anemia (hemoglobin 8.5 g/dL or ferritin 30 ug/L) should use intravenous iron, which appears to be an effective and more secure alternative because oral iron therapy could trigger adverse reactions in them. A meta-analysis has also confirmed that women receiving iron through IV reach their targeted Hb levels more often and quickly with fewer side effects The well-tolerated nature of intravenous iron (IV) is attributed to the existence of type II iron complexes (88).

Due to the higher prevalence of iron deficiency policymakers are discussing whether universal iron supplementation should be adopted as a policy for pregnant women. In this regard, the Office of Dietary Supplements (ODS) of the National Institutes of Health (NIH) recently organized a seminar to evaluate the requirement for iron supplementation treatment among expecting women who already have enough iron (89–91). Hence, further research is needed, before the implementation of this policy (14–16, 92).

There are other interventions to address iron deficiency including iron fortification of staple foods, including wheat, maize, and rice according to the dietary habits of the affected population group is regarded as the most cost-effective long-term approach to reduce the prevalence of anemia especially in low-income countries (93, 94). The final possible strategy for anemia management is nutritional counseling. According to the systematic review, a study in which only counseling was used turned out to be effective in anemia prevention and management (95). The World Health Organization advises pregnant women to get counseling about healthy eating and keeping physically active during pregnancy (85).

### 2.6 Overconsumption

Moreover, iron supplementation can cause some undesirable effects, like oral supplementation, which may induce some common gastrointestinal symptoms, including vomiting, bloating, abdominal pain, diarrhea, the presence of blackish or tarry stools, and constipation (17), while the administration of intravenous (IV) iron can lead to adverse effects such as injection site irritation, skin discoloration, general discomfort, and a metallic taste (73).

One study indicated that there is an increase in the risk of gestational diabetes as a result of iron supplementation in non-anemic women. Zhang et al. concluded in a prospective study that more than 30 mg of elemental oral iron daily for more than 3 months periconceptionally increased the risk of gestational diabetes (96); however, meta-analyses have shown that this is not the case (97). Overall, Women should consume individualized iron supplementation doses based on their geographic region anemia prevalence throughout the pregnancy to enhance iron levels and outcomes may serve as a financially effective intervention. This approach would help limit the risks associated with iron excess and its potential adverse effects (42–44, 74).

## 3 Folate

Folate, also referred to as vitamin B9, represents both its natural and synthetic form., Folate plays a vital role in various physiological processes. These processes comprise the production of nucleotides, the repair and methylation of DNA, and protein metabolism (e.g., the breakdown of homocysteine). Pregnant women require 5 to 10 times more folate compared to nonpregnant women due to increased fetal development, placental growth, uterine expansion, and higher blood volume (18–21). The recommended intake value for folate is 500–600 μg/d for pregnant women in different countries (98).

### 3.1 Sources

Hence, it is crucial to ensure that the recommended daily intake of folate for pregnant women must be fulfilled either through dietary sources or through supplementation throughout pregnancy (99). Natural sources of folate include a variety of food items, like green leafy vegetables (e.g., asparagus and broccoli), citrus fruit, legumes, yeast, lima beans, and organ meats (e.g., beef liver) (100, 101).

### 3.2 Absorption and factors affecting absorption

To be absorbed, folates must be converted enzymatically into folate monoglutamates by folate reductase in the jejunal mucosa. In the liver, folate monoglutamate is converted to dihydrofolate (by an enzyme called dihydrofolate synthase) and to tetrahydrofolate (by an enzyme called dihydrofolate reductase). Tetrahydrofolate is converted into 5,10-methylenetetrahydrofolate by serine hydroxymethyltransferase 5 indicated in Figure 1 (102). The absorption of folate from dietary sources is not efficient as 50 to 75 percent of the folate content is lost during the production and processing of food. Folate insufficiency can also arise due to diminished absorption or metabolism, as well as greater requirements or utilization of folate. In this regard, it has been observed that folic acid exhibits a twofold higher ability for absorption in comparison to folate. It has been found that folic acid supplements show 85% bioavailability when consumed with a meal and 100% bioavailability on an empty stomach (101, 103). Moreover, the effect of the food matrix and the type and amount of folic acid consumed also influence folic acid bioavailability as shown in Figure 2 (104). To account for the bioavailability difference between folate and folic acid, scientists developed a way to compare the two, known as daily folate equivalents (DFE). DFEs adjust for the nearly 50 percent lower bioavailability of food folate compared with that of folic acid: 1 μg of dietary folate equivalent = 0.6 μg of folic acid from fortified food or as a supplement taken with meals = 1 μg of food folate = 0.5 μg of a supplement taken on an empty stomach (105).

### 3.3 Deficiency

Pregnant women may exhibit a greater vulnerability to folate insufficiency because of their elevated physiological demand for folate, as epigenetic processes are observed to take place in both the placenta and the fetus, and certain hormones (estrogen and progesterone) exhibit increased concentrations, which in turn reduce the absorption of folate during pregnancy (73, 106, 107). Women of fertile age, especially in underdeveloped countries, are susceptible to folic acid insufficiency (108), particularly where malaria and sickle-cell anemia are common (109), as well as those with inflammatory bowel disease who use alcohol or tobacco or who use antifolates, anti-epileptics, and anti-inflammatory drugs (IBD) (101, 103).

Insufficient folic acid during pregnancy has the potential to adversely affect both maternal and fetal health. In the developing fetus, inadequate folic acid can cause many irreversible adverse effects, such as congenital disorders, fetal growth restriction, and premature infants. Congenital anomalies, sometimes also referred to as birth defects, have the potential to impact many organ systems within the human body. Congenital heart abnormalities (CHDs) and neural tube defects (NTDs) are the predominant classifications of congenital anomalies. Neural tube defects (NTDs) include two unique congenital malformations, specifically spina bifida and anencephaly. Spina bifida is a congenital condition characterized by improper growth of the spinal cord, whereas anencephaly is a congenital anomaly characterized by the lack of brain and cranial structures in a neonate. Both illnesses have a high mortality rate. In mothers, insufficient levels of folic acid can lead to serious difficulties, such as the occurrence of pre-eclampsia, spontaneous miscarriage, and various placental malformations, including abruption (110–113).

### 3.4 Biomarkers

There are two indications utilized in the assessment of folate deficiency: serum folate concentrations and red blood cell (RBC) folate concentrations. Serum folate concentrations are indicative of recent food intake, while red blood cell (RBC) folate concentrations reflect the body’s stores of folate. Serum or plasma folate levels below 4 nanograms per milliliter or 10 nanomoles per Liter and red blood cell folate levels below 151 nanograms per milliliter (ng/mL; or 340 nanomoles per liter, nmol/L) are indicative (114).

### 3.5 Supplementation and dosage

Meta-analysis and systematic review have indicated a significant association between maternal folic acid intake and the risk of congenital anomalies. In particular, children whose mothers took periconceptional folic acid supplementation were 77% less likely to have congenital defects (115). WHO recommends 400 μg (0.4 mg) of folic acid as early as possible during pregnancy (ideally before conception) to prevent neural tube defects (84). Both dosage and timing are crucial factors to consider in folic acid supplementation. This is due to the neural tube closing on day 28 following conception, a period during which pregnancy may not yet be diagnosed as 41% of pregnancies worldwide are unintended. Hence, the initiation of folic acid treatment beyond the first month of pregnancy fails to offer sufficient efficacy in the prevention of neural tube defects (114, 116–118). To address this issue some governments adopted mandatory folic acid fortification of cereal products, mainly flour. Folic acid fortification would prevent approximately 40–50% of NTD cases by raising average plasma folate levels from baseline levels of approximately 5 ng/mL to, say, 20 ng/mL. If the level was at 40 ng/mL, which is achieved by 4 mg of folic acid per day, it would be almost 80% (119). In 2,000 Chile introduced 0.22 milligrams of folic acid per 100 grams of wheat. By doing so the average daily folic acid consumption increased by 0.43 mg a day, resulting in a 43% reduction in NTD risk (120). But after 9 years Chile reduced the fortification level to 0.18 milligrams per 100 gram wheat flour due to theoretical safety concerns and this resulted in a 10% increase in the NTD (121). Moreover, after mandatory folic acid fortification in the United States and Canada, the occurrence of some NTD declined by 28% (122) and 46% (123). However, two studies by Dorise and Beringer found that only 20% (124) and 55% (125) of pregnant women in Australia met the folate recommendations from diet alone (including natural food folate and fortified foods), respectively. The United Nations International Multiple Micronutrient Antenatal Preparation (UNIMMAP) is a well-known multiple micronutrient formula containing 15 vitamins and minerals, containing iron and folic acid in recommended dosages. An analysis conducted by World Health Organization (WHO) finds that UNIMMAP-MMS lowers the risk of small for gestational age (SGA) and low birth weight (LBW) (126). An additional individual patient data (IPD) meta-analysis also finds MMS(multiple micronutrient supplements) lower the risk of stillbirth and preterm birth (127). Overall, food alone (even fortified food) is usually not enough for women to meet their daily folate needs, so supplements are often recommended.

### 3.6 Overconsumption

Folate is regarded as non-toxic due to its water-soluble nature, which enables it to be eliminated from the body by the urinary system (128). Nevertheless, the consumption of folic acid over 1,000 mg/day has been associated with several negative consequences, such as an elevated risk of specific types of cancer, including breast, prostate, and colon cancer (42–44, 101, 129–131). Increased folic acid may also conceal b12 insufficiency, which contributes to megaloblastic anemia and neuropathy Additionally, it has the potential to induce zinc malabsorption, leading to subsequent deficits in immunological, neurological, and gastrointestinal functionality (42–44, 129, 131–133).

Several studies have identified a correlation between periconceptional folic acid (FA) supplementation and increased rates of twin pregnancies and miscarriages. Multiple births are linked to an increased risk of a few prenatal problems as well as infant morbidity and mortality (134). Moreover, folic acid supplementation does not affect the birth weight (135). WHO also recommends multivitamin supplements instead of iron-folic acid supplements alone to improve birth weight (74). Nevertheless, on the balance of benefits and risks, adequate folate intake through supplements and diet is advised for all women during preconception and pregnancy (109) (Table 3).

**Table 3:** The table represents a summary of key details on iron, folic acid, calcium, and iodine for pregnant women.

Nutrient	Importance	RDA for pregnant women	Deficiency risks and impact	At-risk women population	Tolerable upper limit (Ul)	Overconsumption risks	Relevant biomarker
Iron	Synthesis of hemoglobin and myoglobulin for transportation of oxygen (8–10, 39)	27 mg/day (187)	Iron Deficiency Anaemia (IDA) premature delivery, low birth weight, or small for gestational age (SGA) infants (9, 77, 109)	Vegan/vegetarian women, women suffering from IBD. Women of reproductive age (11, 13, 71, 73–75, 198)	45 mg/day (187)	Increased rate of gestational diabetes, metabolic syndrome in mid-pregnancy, elevated blood pressure (257–259)	<110 g/L (1^st^ and 3^rd^ tri) <105 g/L (2^nd^ tri) (76)
Folic Acid	Synthesis of RNA AND DNA and DNA methylation (18–21)	600 (μg/day) (101)	Birth defects (CHDS, NTDS) IUGR, pre-term delivery (110–113)	Women living in malaria and sickle cell disease prevalent areas. Women using antifolates, anti-epileptics, anti-inflammatory drugs (101, 103, 108, 109)	1,000 (μg/day) (101)	Can mask deficiency of b12 Can malabsorption of Zn Can increase the risk of certain cancers (42–44, 129–131)	Serum/Plasma Folate level <4 ng/mL (<10 nmol/L) (114)
Calcium	Bone formation, muscle contraction, hormone and enzyme activity, nerve cell functioning, and maintenance of cell membranes (136, 138)	1,000-1200 mg/day (137, 146, 147)	Pregnancy-induced hypertension, pre-eclampsia, significant loss of fetal bone minerals during the development, and the potential to induce lasting effects such as insulin resistance in offspring (159–163)	Women consuming little to no milk or dairy products, take a diet packed with fiber, vegan, Asian women with low vitamin D status. Women with lactose intolerance or with cow’s milk allergy, amenorrhea, hypochlorhydric stomach, celiac disease, high-performance athletes (142, 158, 165–167)	2,500 mg(pregnant women aged 19 to 50) (173)	Hypercalcemia (can lead to vascular and soft tissue calcification), nephrolithiasis, hypercalciuria, and other major maternal, fetal, and neonatal complications (174)	The normal range for serum calcium is 2.15–2.50 mmoL/L (153)
Iodine	Vital for the biosynthesis of thyroid hormones that play a critical role in regulating growth, development, metabolism, fetal growth, and differentiation (176, 177)	250 μg/day (192)	Maternal and fetal hypothyroidism, isolated hypothyroxinemia, spontaneous abortion, stillbirth, birth cretinism, congenital disabilities, maldevelopment of the fetal brain, and an increased risk of perinatal death (215–218)	Women of Africa and South/South-East Asia, Europe, the USA, Australia, the Republic of Ireland, and the UK Vegans and vegetarians, smokers, and women consuming diets like salt-restricted, low in dairy, Paleolithic or low salicylate diet, overconsumption of processed foods, or any poorly balanced diet (214, 227, 228)	1,100 μg/day (85, 229)	Hyperthyroidism, hypothyroidism, elevated risk of subclinical hypothyroidism and isolated hypothyroxinemia, fetal hypothyroidism, goiter, and a distinct risk factor of macrosomia (36–38, 230–232)	Insufficient (mUIC < 150 μg/L) Adequate (mUIC 150–249 μg/L) Above requirements (mUIC 250–499 μg/L) Excessive (mUIC ≥ 500 μg/L) Free T4 serum concentration 6.75–18.75 μg/dL (206–209)

## 4 Calcium

Calcium makes up 1 to 2% of total body mass and is the most abundant mineral in the body. It is vital for various physiological mechanisms as well as reactions such as bone formation, muscle contracting, hormone, and enzyme activity, and is a crucial intracellular component that maintains cell membranes and serves an imperative part in the functioning of nerve cells (136–138). Calcium demands increase during gestation as women lose stored calcium as a result of fetal skeleton development. Especially during the third trimester, when maximal calcium accretion occurs for rapid mineralizing of the fetal skeleton. Increased intestinal calcium absorption, renal calcium conservation, and mobilization of calcium from the maternal skeleton are the three potential calcium sources to assist fetal bone accretion (74, 133, 139, 140).

### 4.1 Sources

Calcium that is lost during gestation can only be substituted by dietary calcium consumption, foods fortified with either organic or inorganic calcium, and dietary supplements. Calcium citrate and calcium carbonate make up the largest proportion of calcium dietary supplements (133, 141). Milk, dairy goods, green leafy vegetables, fish with soft bones (e.g., sardines), and legumes are the richest dietary sources of calcium. For vegans, nuts, dried fruits, fortified soy milk, and soy products like tofu are excellent substitute dietary options for calcium (142).

### 4.2 Absorption and factors affecting absorption

There are two general mechanisms by which ingested calcium is absorbed by mammalian small intestines: transcellular active transport in the duodenum and upper jejunum, and paracellular passive transport throughout the intestine (143, 144). Cow’s milk and its derivatives are the highest-quality and most bioavailable sources of calcium. The calcium bioavailability of other animals’ milk, soy milk, and certain vegetables is comparable with that of cow’s milk. Some foods might have higher concentrations of calcium, yet their bioavailability fluctuates, as phytates and oxalates are absorbed less efficiently than carbohydrates. Vitamin D, carbohydrates, particularly lactose from milk and dairy products, L-lysine, L-arginine and ascorbic acid (vitamin C) have a synergistic impact on the uptake of calcium, whereas fiber-rich foods, fats, phosphates, protein (not completely understood), caffeine, and alcohol have an opposing effect as shown in Figure 2 (143, 145).

### 4.3 Dosage and supplementation

Calcium requirements range between 1,000 and 1,200 mg/day (146, 147). There is a rise in calcium needs during gestation which can be met via dietary intake however, taking calcium supplements at doses between 300 mg and 2,000 mg per day has been suggested (136). From the start of the 20th week of gestation until the end of pregnancy, pregnant women are recommended to get 1.5–2.0 g of mineral calcium per day if they are at elevated risk or in conditions where there is insufficient dietary intake of calcium. The total everyday amount should be split into three doses, ideally during meals (137, 148).

A complete prenatal calcium supplementation program that was piloted in the Dailekh area of Nepal was assessed in one study. In order to distribute and advise pregnant women on the use of calcium through government antenatal care (ANC) programs, the initiative trained medical professionals and community health volunteers. 94.6% of pregnant women in the district were covered by the calcium distribution through ANC, according to a survey of 1,240 recent postpartum women. To receive the entire 150-day course of calcium supplements, the majority of women (more than 80%) attended ANC early. Pregnant women reported high levels of acceptability, compliance (79.5% consumed the entire allowance), and coverage; almost all said they would be ready to recommend the calcium and use it in future pregnancies. The program also maintained consistent calcium availability, suggesting this universal free supplementation model can be successfully scaled up in other parts of Nepal (149).

It should be noted that adequate vitamin D status [circulating level of 25(OH)D being >80 nmol] in the body is critical for the regulation of intestinal calcium absorption and bone homeostasis. Also, vitamin K is necessary for the activation of Gla proteins which are also responsible for the regulation of calcium in the body (150–152).

### 4.4 Biomarkers

Serum calcium, parathyroid hormone (PTH), and 1,25(OH)2D levels are essential biomarkers for determining calcium levels in the human system. 2.15–2.50 mmoL/L is considered to be the standard range for serum calcium. A rise in the concentrations of PTH and 1,25(OH)2D suggests that the body needs greater calcium absorption (153–156).

### 4.5 Deficiency

In 2011, about 51% of the global population, or approximately 3.5 billion individuals, were vulnerable to calcium deficiency, with 90% of this population residing in Africa and Asia. Even in High-Income nations, several demographics fail to comply with the recommendations (157, 158). Calcium deficiency during gestation can impact both the mother and fetus. Maternal calcium shortages during gestation have the potential to cause long-lasting impacts, such as insulin resistance in offspring. It is possible through the modification of many metabolic characteristics and the epigenetic control of gene expression (159). Additionally, it can result in pre-eclampsia, pregnancy-induced hypertension in mothers, and a considerable loss of bone minerals in the developing fetus (160–163).

There are several strategies that can be used to assist increase calcium intake. These include encouraging the intake of foods that are naturally high in calcium, fortifying staple foods, producing more calcium-containing crops through biofortification, and employing food processing methods that can increase calcium content or bioavailability. Policymakers who want to increase calcium intake can use these interventions. It is highly recommended that the coverage and health effects of such treatments be tracked and evaluated (164).

### 4.6 Women at risk

Women who ingest minimal to no milk or dairy products, vegans, high-performance athletes, Asian women having inadequate vitamin D status, those who take a high fiber diet, have allergies to cow’s milk or are lactose intolerant, have conditions like hypochlorhydric stomach, or have celiac disease are at risk of being calcium deficient (142, 158, 165–167). Additionally, a meta-analysis has demonstrated that in Low and middle-income countries, the dietary calcium intake during pregnancy is very low, unlike the High-income countries (25, 168). Low calcium intakes across Asian, African, and Latin American countries have been reported in a review (169, 170). The average intake of calcium in low- and middle -income countries was found to be 648 mg/day, whereas that from high-income countries was 948 mg/day (171, 172).

### 4.7 Overconsumption

Like deficiency, overconsumption of calcium can also seriously affect both maternal and fetal health. For pregnant women between the ages of 19 and 50, the tolerable upper intake level (UL) of calcium is 2,500 mg. Calcium overconsumption can cause hypercalcemia (rare), which can lead to vascular and soft tissue calcification, nephrolithiasis, hypercalciuria (which also happens naturally during gestation), and other major maternal, fetal, and neonatal problems (24, 173, 174).

## 5 Iodine

Iodine is a trace mineral that accounts for 0.00004% of the total human weight and is most concentrated in the thyroid gland, muscle, and numerous endocrine tissues (175). In thyroid glands, iodine serves as vital for the biogenesis of thyroid hormones that are necessary for the optimum functioning of almost all tissues and perform a crucial role in the regulation of growth, development, differentiation, and metabolic processes, significantly influencing the body’s metabolic activity and utilization of oxygen. Within the uterus, they are essential for fetal growth and differentiation (176, 177). Since there is an upsurge in hormone demands commencing in the initial 13 weeks of pregnancy, pregnant women experience dramatic changes in thyroid function and a hike in iodine needs (31, 178).

### 5.1 Sources

The primary source of iodine for humans is diet, although certain populations obtain it from water. Seaweed, marine-sourced fish and shellfish, eggs (particularly fortified), dairy products, livestock, and iodized salt are iodine-rich food sources. However certain categories of fish and seafood pose an elevated risk of contamination with parasitic organisms, pathogens, and toxic substances. Safe processing and proper handling can reduce this risk (179–185).

### 5.2 Absorption and factors affecting absorption

There are few studies regarding the bioavailability of iodine from diet, and these imply that the actual bioavailable quantity tends to be less than what is consumed. Upon being converted to iodide, the gastrointestinal tract swiftly absorbs iodine from food (186). Upon entering the circulation, iodide is absorbed in appropriate amounts by the thyroid gland and excreted in most amounts in the urine (187). However, the absorption efficacy by the gastrointestinal tract of consumed iodine is regarded as high (greater than 90%) as shown in Figure 1 (188). Seaweed contains abundant iodine concentrations. *In vitro* studies evaluating iodine absorption in different seaweeds have found that 49–82% of the iodine can be absorbed during digestion in the gut (189). Pasteurization has been shown to reduce the iodine level in milk, while sterilization has no impact on the iodine concentrations in milk (179–183). It has been shown that goitrogens in cruciferous vegetables and soy products, can interfere with the synthesis of thyroid hormones in a variety of ways, primarily by inhibiting the utilization of iodine as indicated in Figure 2 (190).

### 5.3 Dosage and supplementation

As a result of maternal thyroid stimulation, iodine requirements increase by around 50 percent during pregnancy, leaving both the mother and the developing fetus susceptible (181, 191). The 2007 recommendations of the World Health Organization (WHO), the United Nations Children’s Fund (UNICEF), and the International Council for the Control of Iodine Deficiency (ICCIDD) recommend a daily iodine consumption of 250 mcg during gestation (192). In iodine-deficient regions, controlled studies have shown that iodine supplemental intake before or throughout early gestation eradicates emerging instances of cretinism, increases birth weight, and mitigates the incidence of perinatal and infant fatalities. Also, it enhances developmental outcomes in youngsters by 10 to 20% (193).

A randomized, double-blind trial in Sweden found that providing a daily 150 μg iodine supplement to mildly iodine-deficient pregnant women improved their iodine status. The intervention group reached iodine sufficiency, with median urinary iodine concentrations (UICs) of 139 μg/L and 136 μg/L in the second and third trimesters, respectively. The intervention group also exhibited higher median UICs and lower median thyroglobulin levels compared to controls, without affecting other thyroid markers or neonatal outcomes (194).

There are two primary methods for administering supplemental iodine: daily administration of potassium iodide or yearly administration of a gradually released iodine formulation like iodized oil (195). Because the thyroid is capable of storing iodine, it appears that iodine before pregnancy is as essential as, if not more essential than, iodine during gestation (196). A cohort study found that lower iodine status during pregnancy and postpartum was associated with lower TSH and higher fT3 and fT4 concentrations. Initiating iodine-containing supplements before and during pregnancy was associated with more optimal thyroid function compared to no supplement use. These results support the importance of optimizing iodine intake before pregnancy (197). Iodine supplemental intake in the form of multivitamins potassium iodide containing 150 μg of iodine is prescribed as early as the onset of pregnancy and even sooner in planned pregnancies (198–201).

In 2009, Australia introduced mandatory iodized salt fortification in bread to address mild iodine deficiency. A study analyzed the program’s impact on iodine intake using 2011–2012 national dietary data, finding that the fortification effectively achieved adequate iodine levels across socioeconomic and geographic groups, with significant benefits for women of childbearing age and children (202).

### 5.4 Biomarkers

Serum iodine concentration (SIC) and thyroglobulin (Tg) are important iodine metabolism biomarkers. Urinary iodine is also an accurate marker for recent iodine consumption as urinary iodine excretion indicates over 90 percent of dietary iodine intake in a constant state (203–205). For Median urinary iodine concentration (mUIC), in 2007, WHO, UNICEF, and ICCIDD updated the epidemiological guidelines of iodine levels for women during pregnancy and suggested the general public median UIC value as the assessing marker having an adequate level range of 150–249 g/L (206, 207). Serum levels of thyroid hormones are measured to evaluate the thyroid’s functioning and iodine status. Total T4 (TT4) determination is preferable over free T4 (FT4) determination, particularly during the final stages of gestation. The adjustment of the TT4 in the gestational period by a factor of 1.5 relative to nonpregnant benchmarks gives a practical FT4 measurement. For actual practice, the nonpregnant baseline range of 4.5–12.5 g/dL will change to 6.75–18.75 g/dL (208, 209).

### 5.5 Deficiency

Nearly 2 billion individuals worldwide are suffering from iodine deficiency (ID), with roughly 50 million exhibiting medical symptoms (210). Overall, 53% of the pregnant population has insufficient iodine intake (211). The insufficiency of iodine is widespread in the majority of countries. Among these countries, Africa and Southeast Asia are the most affected (212). Iodine deficiency also places some developed countries at risk, including continental Europe, the United States, Australia, the Republic of Ireland, and the United Kingdom (213). For women of reproductive age, appropriate iodine levels are crucial. It must be taken into account that not only severe prenatal deficient levels of iodine but also minimal to significant shortfalls are all linked to negative effects arising in the progeny (214).

As soon as the daily intake of iodine declines below 100 μg, iodine deficiency during gestation gets usually serious (195). Iodine deficiency during pregnancy may cause low thyroid hormone levels in both mother and fetus, leading to birth complications and impaired brain development in the baby, increasing the risk of perinatal death (215–218). Additionally, there is mounting evidence that iodine deficiency in pregnancy has an adverse and irreversible effect on the neurocognitive development of the embryo, particularly when the shortage occurs during the first trimester (219–222). A cohort study investigated the impact of maternal iodine status on offspring cognitive outcomes. Mother–child pairs from the Avon Longitudinal Study of Parents and Children (ALSPAC) cohort were examined by assessing urinary iodine content and creatinine to adjust for urine volume in preserved samples from 1,040 first-trimester pregnant women. Women were chosen based on having a singleton pregnancy and the availability of both a urine sample from the first trimester (defined as ≤13 weeks’ gestation; median 10 weeks [IQR 9–12]) and an intelligence quotient (IQ) assessment of the offspring at 8 years of age. The iodine-to-creatinine ratios in women were classified as <150 μg/g (deficient) or ≥ 150 μg/g (adequate) according to WHO pregnancy standards.

Mild-to-moderate iodine deficiency was observed in the cohort (median urinary iodine: 91.1 μg/L). Maternal iodine-to-creatinine ratios <150 μg/g were associated with increased odds of low verbal IQ, reading accuracy, and comprehension scores in children. A dose–response relationship was evident, with cognitive outcomes worsening as iodine levels decreased. Results suggested that even mild-to-moderate iodine deficiency during pregnancy may adversely affect children’s cognitive development, highlighting the importance of adequate iodine nutrition in pregnancy (223).

However, no effects were found on preterm birth, low birth weight, and hypertensive disorders among euthyroid pregnant women with different urinary iodine concentrations (UIC). Another systematic review confirmed no effects of maternal UIC on anthropometric measures of new born. Moreover, no link was found between maternal urinary iodine and the brain morphology of the infant (224–226).

### 5.6 Women at risk

Deficiency of iodine can result from restricted salt intake as a preventative measure against numerous noncommunicable diseases, a Paleolithic or low salicylate diet, an eating pattern with generally reduced dairy intake, an excessive intake of industrial foods that may not have iodized salt, or any poorly balanced diet (214). Due to the limited iodine quantity found in plant-based foods, vegans and vegetarians are also vulnerable to iodine deficiency (227). Moreover, smoking and iodine exhibited a statistically significant negative interaction (228).

### 5.7 Overconsumption

Likewise, excessive iodine consumption during the gestational period may result in adverse outcomes. The upper intake level of iodine is 1,100 mcg/day (85, 229). There is a potential for adverse consequences associated with excessive consumption of iodine during pregnancy, and the suggested UI of iodine consumption is disputed. Ingestion of iodine supplementary products, water, diet, or medication containing an excessive amount of iodine, as well as exposure to objects having iodine, like radiological contrast material and disinfecting agents, might result in more than iodine. Hyperthyroidism, hypothyroidism, an increased risk of subclinical hypothyroidism, and an elevated risk of isolated hypothyroxinemia may result from abundant iodine (36–38). Along with causing fetal hypothyroidism and goiter and being a distinct risk factor for macrosomia, too much iodine consumption may be associated with additional negative pregnancy outcomes (230–232) (Table 2).

## 6 Conclusion

Pregnancy is the most crucial stage in a woman’s life, and diet during this period determines the health of the mother and newborn. Ideal micronutrient intake is often neglected during this time, as the major focus is often on the increased calorie requirements. Micronutrient deficiencies can lead to some serious health issues for the mother and fetus and also increase the fetal mortality risk. This review covers all aspects related to the consumption of four major micronutrients iron, folic acid, iodine, and calcium during pregnancy including their dietary sources and factors that affect their bioavailability. Ideally, their requirements should be met by dietary sources but their increased requirements during pregnancy demand other means, like the use of supplements. These supplements must be given according to the individual needs and RDAs set by the WHO. Because not only deficiency but also overconsumption has serious health implications. This emphasizes the need for a thorough nutritional assessment of pregnant women which helps decide whether there is a need to take supplements or whether the requirements can be fulfilled by dietary sources.

This review helps the readers to understand the importance of sufficient micro-nutrient intake for a healthy gestation period. To increase awareness, there must be educational programs and training for not only pregnant women but all women of reproductive age on maintaining the ideal body weight and eating for a healthy future generation. More programs should be designed for populations that are at risk of deficiency, like introducing supplemented or fortified foods. Cost, access, and availability are important factors to be considered before implementing an intervention in underdeveloped or developing countries.

There is a lack of studies with multiple micronutrient supplements that evaluate their combined effects, including synergistic and antagonistic interactions between them as well as the underlying mechanisms of these interactions. Furthermore, for pregnant women taking certain medications like blood-thinning medicines, we need to study in detail the effects of medicines on micronutrients’ bioavailability and other pregnancy outcomes. It is also crucial to do research on pre-conceptional diet and set RDAs for women trying for conception. Moreover, samples used in these studies should be alike to avoid variations in results due to various factors like the age of the mother at the time of conception, weight before and during pregnancy, gap between successive pregnancies, multiple pregnancies, presence of any infertility syndrome like polycystic ovarian syndrome and family history of congenital disorders. It is also required to study twin or triplet pregnancies separately to set RDAs for women having multiple gestation. Hence, to date, there are a lot of knowledge gaps to cover to gain a proper understanding of micronutrient intake during pregnancy.
